# Cultural and Institutional Considerations in Advance Care Planning in Long-Term Care Settings

**DOI:** 10.1093/geroni/igaa057.2716

**Published:** 2020-12-16

**Authors:** Gloria Gutman, Brian de Vries

## Abstract

Advance Care Planning (ACP) is a process that supports individual’s understanding and sharing of personal values, life goals, and preferences regarding future medical care, so that they obtain care consistent with these during serious and chronic illness. While ACP is important for all, it is especially so for people who fall outside traditional, western, heteronormative contexts (e.g. who belong to ethnic, racial and/or sexual/gender minorities). This symposium draws from research conducted by the Diversity Access Team [part of a national project iCAN-ACP Improving Advance Care Planning for Frail Elderly Canadians]. The first paper presents results from focus groups conducted with loved ones of South Asian, Chinese and Lesbian, Gay, Bisexual, and Transgender (LGBT) older adults living in care homes; issues identified as barriers include starting ACP conversations too late (“my husband has severe dementia”), lack of consideration of cultural traditions and, in the case of LGBT older adults, their non-family support networks. The second paper draws from focus groups with care home staff, implicating their own training as a barrier to assisting residents/families with ACP as well as resident, family, institutional and cultural influences. A third paper reports on an educational intervention designed to increase staff understanding of ACP and comfort in assisting residents/families with ACP. The fourth paper reports feedback received on two ACP planning tools, reflecting the importance of minority group representation in visuals and text. Together, these papers underscore the importance of taking culture into consideration in framing and discussions of fostering ACP among minority populations.

